# A pretreatment prediction model of grade 3 tumors classed by the IASLC grading system in lung adenocarcinoma

**DOI:** 10.1186/s12890-023-02690-3

**Published:** 2023-10-07

**Authors:** Kai Wang, Xin Liu, Yun Ding, Shuai Sun, Jiuzhen Li, Hua Geng, Meilin Xu, Meng Wang, Xin Li, Daqiang Sun

**Affiliations:** 1 Clinical School of Thoracic, Tianjin Medical University, Tianjin, China; 2 Department of Thoracic Surgery, Tianjin Chest Hospital, Jinnan District, No. 261, Taierzhuang South Road, Tianjin, 300222 China; 3 Department of Pathology, Tianjin Chest Hospital of Tianjin University, Tianjin, China

**Keywords:** Lung adenocarcinoma, Lobectomy, IASLC grading system, Prediction model, Nomogram

## Abstract

**Purpose:**

The new grading system for invasive nonmucinous lung adenocarcinoma (LUAD) in the 2021 World Health Organization Classification of Thoracic Tumors was based on a combination of histologically predominant subtypes and high-grade components. In this study, a model for the pretreatment prediction of grade 3 tumors was established according to new grading standards.

**Methods:**

We retrospectively collected 399 cases of clinical stage I (cStage-I) LUAD surgically treated in Tianjin Chest Hospital from 2015 to 2018 as the training cohort. Besides, the validation cohort consists of 216 patients who were collected from 2019 to 2020. These patients were also diagnosed with clinical cStage-I LUAD and underwent surgical treatment at Tianjin Chest Hospital. Univariable and multivariable logistic regression analyses were used to select independent risk factors for grade 3 adenocarcinomas in the training cohort. The nomogram prediction model of grade 3 tumors was established by R software.

**Results:**

In the training cohort, there were 155 grade 3 tumors (38.85%), the recurrence-free survival of which in the lobectomy subgroup was better than that in the sublobectomy subgroup (*P* = 0.034). After univariable and multivariable analysis, four predictors including consolidation-to-tumor ratio, CEA level, lobulation, and smoking history were incorporated into the model. A nomogram was established and internally validated by bootstrapping. The Hosmer–Lemeshow test result was χ^2^ = 7.052 (*P* = 0.531). The C-index and area under the receiver operating characteristic curve were 0.708 (95% CI: 0.6563–0.7586) for the training cohort and 0.713 (95% CI: 0.6426–0.7839) for the external validation cohort.

**Conclusions:**

The nomogram prediction model of grade 3 LUAD was well fitted and can be used to assist in surgical or adjuvant treatment decision-making.

**Supplementary Information:**

The online version contains supplementary material available at 10.1186/s12890-023-02690-3.

## Purpose

Although the existing classification of invasive nonmucinous lung adenocarcinoma (LUAD) is closely related to prognosis, the formal grading system was not conclusive until a new grading system was proposed by the International Association for the Study of Lung Cancer (IASLC) pathology committee and finally included in the 2021 World Health Organization (WHO) Classification of Thoracic Tumors [1, 2]. Different from the 2015 WHO Classification, the IASLC grading system classified the cribriform pattern and the fused gland pattern as complex gland patterns and added them to high-grade patterns [1, 3]. Moreover, LUAD was divided into grade 1, grade 2 and grade 3 representing well-differentiated, moderately differentiated and poorly differentiated LUAD on the basis of high-grade components and predominant histologic patterns [1, 2, 4].

According to the latest standard, high-grade patterns were divided into micropapillary patterns, solid patterns, cribriform patterns and complex gland patterns [2]. Since various studies have confirmed the close relationship between high-grade patterns and poor prognosis. The lack of recognition of complex glandular components increased the heterogeneity of the old grading system. Thus, the underestimation of the influence of high-grade components on prognosis was corrected in the IASLC grading system, greatly improving the prognostic discrimination ability [5–8]. In addition, many studies have confirmed that grade 3 tumor was an independent risk factor for poor prognosis of resectable LUAD. It could indicates that grade 3 adenocarcinomas with the most high-grade components should mainly be identified and properly managed [9–12].

Surgery is widely accepted as the optimal treatment choice for clinical stage I (cStage-I) non-small cell lung cancer [13]. At present, many retrospective studies have shown that the prognosis of lobectomy for early LUAD with high-grade components was better than that of sublobectomy. But the correlation between pathological grades and preoperative treatment decisions has not been established [14, 15]. Furthermore, the limited evaluation ability of preoperative small diagnostic samples and intraoperative frozen section analysis to assess pathological grades makes it difficult to assist in decision-making for both surgical and nonsurgical treatments [16, 17]. Therefore, the purpose of this study was to establish a prediction model for patients with cStage-I lung cancer. The model can identify grade 3 high-risk groups, and recommend the optimal surgical and nonsurgical treatment schemes individually.

## Methods

### Patients

**Fig. 1:**
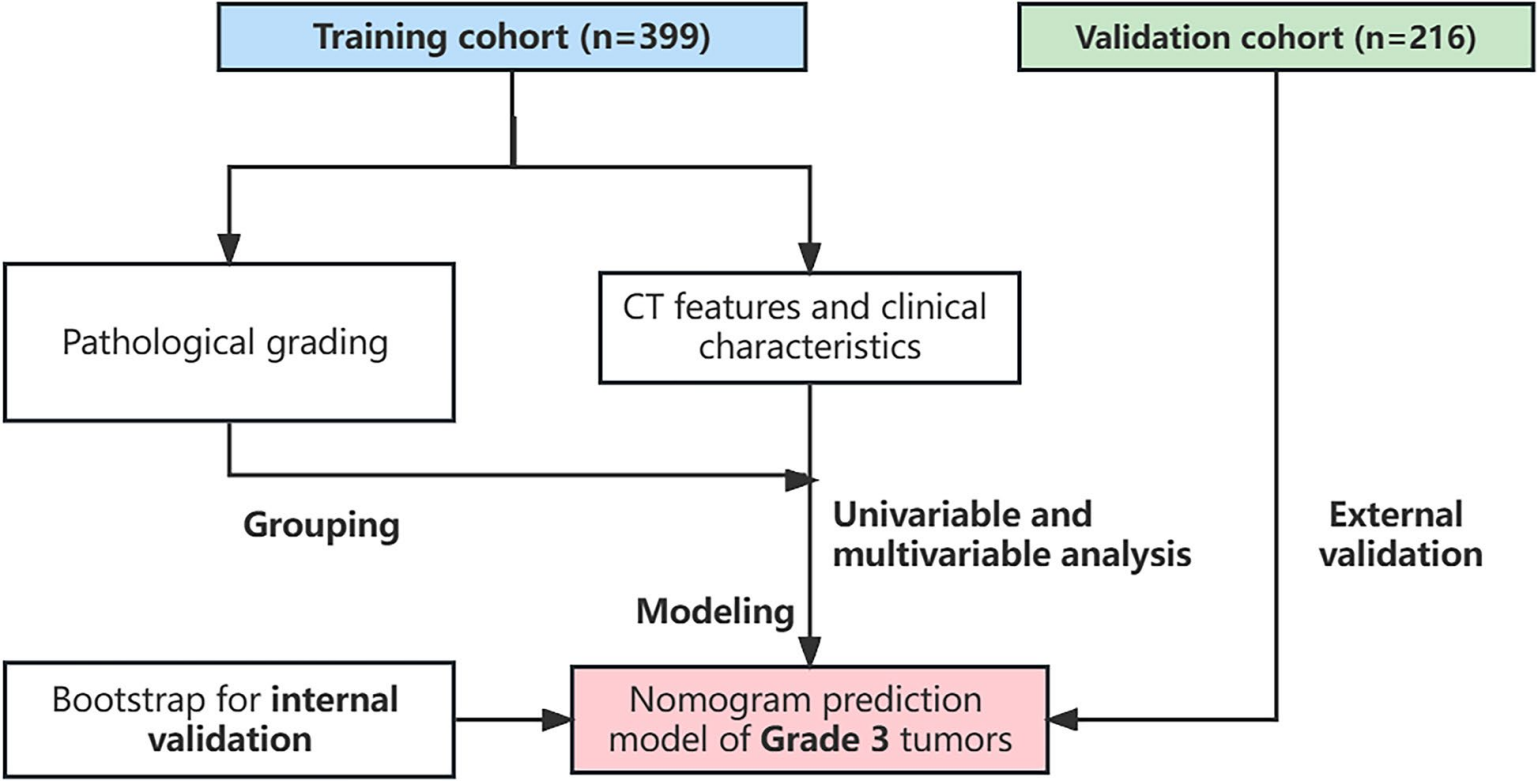
Flow diagram of the enrollment of training and validation cohort and process of model establishment and verification

We retrospectively collected patients with cStage-I nonmucinous invasive LUAD who underwent surgery at Tianjin Chest Hospital from January 2015 to December 2020. The following inclusion criteria were applied: (i) patients with a preoperative diagnosis of clinical stage I LUAD and (ii) underwent complete resection and had pathologically confirmed nonmucinous invasive LUAD. The exclusion criteria were as follows: (i) patients receiving preoperative treatments; (ii) patients with multiple primary tumors; and (iii) lost to follow-up. Ultimately, 399 patients from 2015 to 2018 were included in the training set, and 216 patients from 2019 to 2020 were included as the validation set (Fig. 1). Relevant clinical data were collected from all enrolled patients. The study was approved by the Ethics Committee of the Department of Thoracic Surgery, Tianjin Chest Hospital. Moreover, all patients upon admission signed the consent form for scientific research and the use of clinical data.

### Clinical characteristics

Based on the existing relevant research and the indicators included in the database of our hospital, the clinical information collected in this study was age, sex, preoperative symptoms, smoking history, carcinoembryonic antigen (CEA) level, tumor location and surgical approach. Moreover, the positive preoperative symptoms included cough, expectoration, and hemoptysis. Positive smoking history was defined as smoking at least 1 cigarette per day continuously or cumulatively for a period of 6 months before the clinical check, regardless of whether smoking has ceased. CEA > 4.7 ng/mL was defined as positive. Surgical methods were divided into lobectomy and sublobectomy, of which sublobectomy included wedge resection and segmentectomy. Imaging information included maximum tumor diameter (Tdmax), consolidation-to-tumor ratio (CTR), spiculation, lobulation, pleural traction, air bronchogram and vacuole. CTR was defined as the ratio of the largest diameter of the solid components to the largest diameter of the lesion over the lung windows via CT imaging. Pathological information included pathological types, pathological stages and pathological subtypes. In addition, the 8th edition of the American Joint Committee on Cancer TNM Classification was used for clinical and pathological staging.

### Histologic evaluation

The hematoxylin and eosin-stained sections of surgical specimens of the patients in the training set and the validation set were reviewed and graded according to the IASLC grading system, which combined high-grade patterns and predominant histologic patterns as the grading standards and classified tumors into 3 grades as follows: grade 1, lepidic predominant tumors with no or less than 20% of high-grade patterns (solid, micropapillary, and/or complex glandular patterns); grade 2, acinar or papillary predominant tumors with no or less than 20% of high-grade patterns; and grade 3, any tumor with 20% or more of high-grade patterns. According to the 2015 WHO Classification of Thoracic Tumors, pathological subtypes of invasive LUAD were semiquantitatively estimated. The percentage of histological pattern was recorded in 5% increments. In this study, grade 1 and grade 2 patients were classified into the non-grade 3 group and compared to the grade 3 group. In addition to the IASLC grading system, patients were also graded according to the architectural grading system, which was divided into low grade (lepidic predominant), intermediate grade (acinar and papillary predominant), and high grade (solid and micropapillary predominant).

### Follow-up

Patients in the training set were followed up by telephone, and survival information was recorded on spreadsheets. The primary outcomes were overall survival (OS) and recurrence-free survival (RFS). In addition, OS was defined as the time from surgery to death from any cause, and RFS was defined as the time from surgery to the determination of recurrence. Patients were censored at the last follow-up in September 2021 if no events were recorded.

### Statistical analysis

IBM SPSS statistics 25 and R software 4.1.3 were used for statistical analysis. Survival curves were plotted using the Kaplan‒Meier (K-M) method, while intergroup differences were examined using the log-rank test. Categorical variables are described as frequencies (percentages), and continuous variables are expressed as medians (quartile ranges). The Mann‒Whitney U test, T test, Chi-square test or Fisher exact test were used to compare differences between groups. Univariate and multivariate logistic regression analyses were performed on the training cohort. The indicators with *P* < 0.05 in the univariate analysis were included in multivariate analysis, and the variables with *P* < 0.05 in multivariate analysis were included in the final model. The rms package of R software was used for modeling. Internal validation was performed using bootstrapping, and the original dataset was resampled and repeated 1,000 times to obtain a calibration curve. The area under the receiver operating characteristic curve (AUC) was used to evaluate the predictive ability of the nomogram prediction model, and the concordance index (C-index) was used to assess the discrimination of the model. The model goodness of fit was evaluated using the Hosmer‒Lemeshow test, while clinical utility was evaluated by decision curve analysis (DCA). Differences were considered statistically significant when the *P* value was < 0.05.

## Results

### Clinicopathologic characteristics

Pathological assessments for the training and validation sets are presented in Table 1. According to the IASLC grading system, there were 180 cases (45.1%) of grade 1, 64 cases (16.1%) of grade 2, and 155 cases (38.8%) of grade 3 in the training set. In addition, there were 101 cases (46.8%) of grade 1, 46 cases (21.3%) of grade 2, and 69 cases (31.9%) of grade 3 in the validation set. The baseline features of the training set and validation set are shown in Supplementary Table 1.

**Table 1:** Pathological assessments for the training and validation set

Grading System	Training set (*n* = 399)	Validation set (*n* = 216)
IASLC Grading System, n (%)		
Grade 1	180(45.1)	101(46.8)
Grade 2	64(16.1)	46(21.3)
Grade 3	155(38.8)	69(31.9)
Architectural Grading System, n (%)		
Low grade	245(61.4)	127(58.8)
Intermediate grade	118(29.6)	72(33.3)
High grade	36(9)	17(7.9)
Predominant Subtype, n (%)		
Lepidic	274(68.7)	142(65.7)
Papillary	23(5.8)	29(13.4)
Acinar	76(19)	32(14.8)
Micropapillary	6(1.5)	8(3.7)
Solid	20(5)	5(2.3)

### Correlation between surgical extent and prognosis

**Fig. 2:**
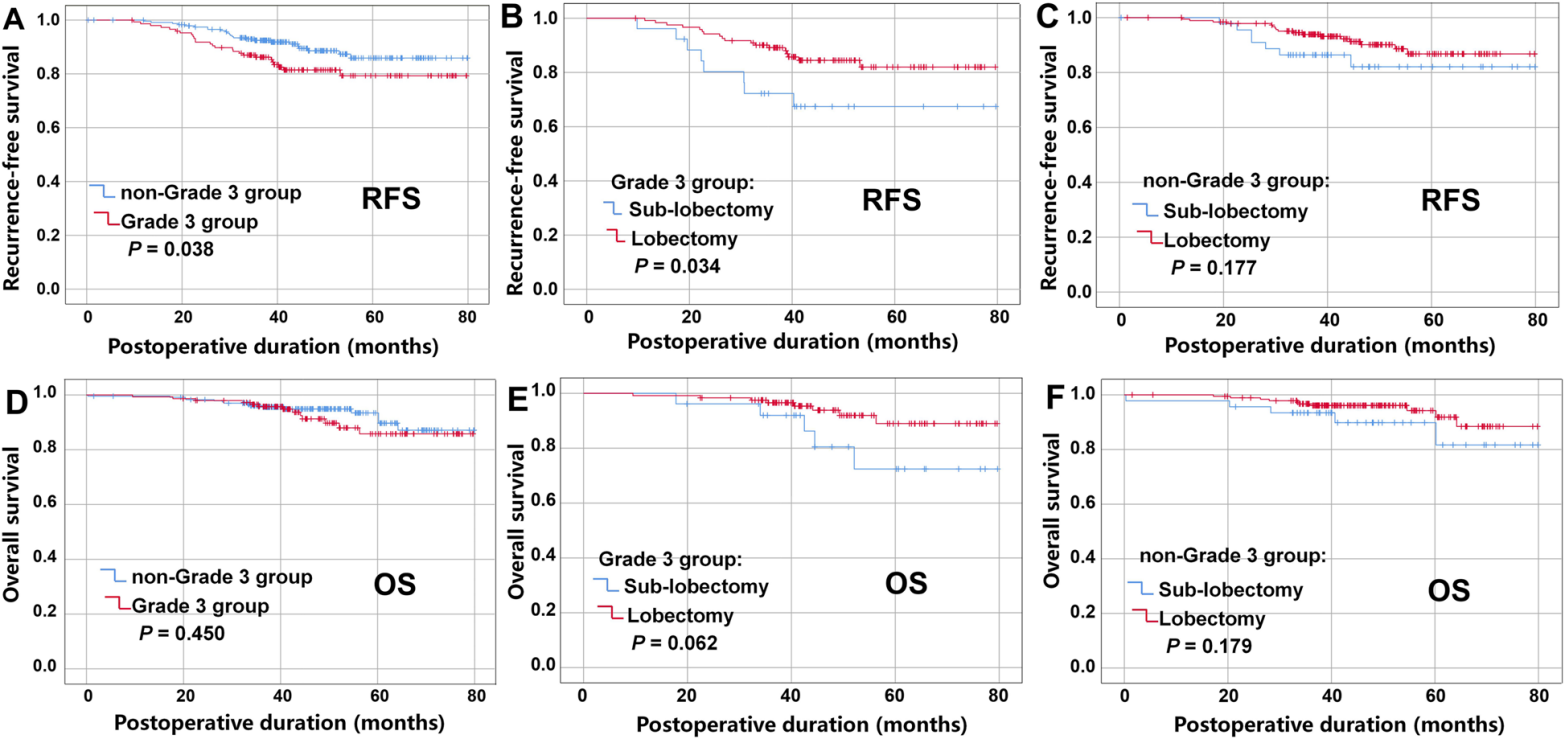
Survival analysis of grade 3 and non-grade 3 tumors in the training cohort using K-M method

In the training set, a total of 127 patients (81.9%) underwent lobectomy, and 28 (18.1%) underwent sublobectomy in the grade 3 group. Additionally, a total of 194 patients (79.5%) underwent lobectomy, and 50 (20.5%) underwent sublobectomy in the non-grade 3 group. The median follow-up time of the 399 patients was 45.7 months (range: 1.5, 80.0). Significantly worse 5-year RFS rates were observed in the grade 3 group compared to the non-grade 3 group (79.3% vs. 85.9%, *P* = 0.038, Fig. 2a). Subgroup survival analysis indicated a significantly better RFS rate in grade 3 patients who underwent lobectomy compared to those who underwent sublobectomy (5-year RFS rate 82.0% vs. 67.4%, *P* = 0.034, Fig. 2b). Conversely, there was no significant difference in RFS between the lobectomy group and the sublobectomy group in the non-grade 3 group (5-year RFS rate 86.8% vs. 82.1%, *P* = 0.177, Fig. 2c). Furthermore, no significant differences were observed in 5-year OS between grade 3 and non-grade 3 groups (*P* = 0.45, Fig. 2d). Similarly, no significant difference in 5-year OS was observed between the lobectomy group and the sublobectomy group for both grade 3 and non-grade 3 groups (all *P* > 0.05), as depicted in Fig. 2e and f.

### The risk factors of grade 3 LUAD

Univariable analysis showed that CTR, lobulation, CEA level, smoking history, and Tdmax were associated with grade 3 tumors (*P* < 0.05, see Table 2). All the above variables were included in the multivariate logistic regression, and the results showed that CTR, CEA level, lobulation and smoking history were independent risk factors for grade 3 tumors (*P* < 0.05, see Table 3).

**Table 2:** Univariable analysis of grade 3 tumors in the training set (*n* = 399)

Variable	Grade 3	Non-grade 3	OR	95%CI	*P*-Value
Age (years), median (P25, P75)	62.000(56.000,67.000)	63.000(58.000,67.000)	0.992	0.967–1.017	0.315
Gender, n (%)					
Male	78(50.323)	105(43.033)	1.341	0.895–2.009	0.155
Female	77(49.677)	139(56.967)	1		
Preoperative symptoms, n (%)					
Yes	37(23.871)	49(20.082)	1.248	0.769–2.025	0.37
No	118(76.129)	195(79.918)	1		
Smoking history, n (%)					
Yes	60(38.710)	63(25.820)	1.815	1.178–2.795	0.007
No	95(61.290)	181(74.180)	1		
CEA level (ng/ml), n (%)					
Positive (> 4.7)	45(29.032)	39(15.984)	2.15	1.321–3.501	0.002
Negative (0–4.7)	110(70.968)	205(84.016)	1		
Tumor location, n (%)					
Central	5(3.226)	6(2.459)	1.322	0.397–4.409	0.649
Peripheral	150(96.774)	238(97.541)	1		
Lobular location, n (%)					
Middle/Lower lobe	67(43.226)	98(40.164)	1.134	0.754–1.706	0.545
Upper Lobe	88(56.774)	146(59.836)	1		
Tdmax (cm), median (P25, P75)	2.300(1.774,2.783)	2.059(1.537,2.536)	1.425	1.079–1.883	0.013
CTR, median (P25, P75)	0.772(0.586,0.974)	0.559(0.364,0.810)	9.482	4.206–21.377	< 0.001
Spiculation, n (%)					
Yes	95(61.290)	141(57.787)	1.157	0.767–1.745	0.488
No	60(38.710)	103(42.213)	1		
Pleural retraction, n (%)					
Yes	101(65.161)	140(57.377)	1.389	0.916–2.107	0.122
No	54(34.839)	104(42.623)	1		
Vacuole, n (%)					
Yes	51(32.903)	64(26.230)	1.379	0.888–2.142	0.152
No	104(67.097)	180(73.770)	1		
Lobulation, n (%)					
Yes	98(63.226)	109(44.672)	2.129	1.409–3.217	< 0.001
No	57(36.774)	135(55.328)	1		
Air bronchogram, n (%)					
Yes	39(25.161)	67(27.459)	0.888	0.561–1.406	0.613
No	116(74.839)	177(72.541)	1

**Table 3:** Multivariable analysis of grade 3 tumors in the training set (*n* = 399)

Variable	*β*	SE	Wald χ^2^	*P*-Value	OR	95%CI
CTR	2.104	0.433	21.599	< 0.001	7.49	3.204–17.509
Lobulation	0.655	0.226	8.389	0.004	1.926	1.236–3.001
CEA level	0.852	0.267	10.155	0.001	2.345	1.388–3.96
Smoking history	0.522	0.235	4.941	0.026	1.685	1.064–2.67
Tdmax	0.034	0.415	0.007	0.936	1.034	0.458–2.334

### Development and validation of the nomogram prediction model

**Fig. 3:**
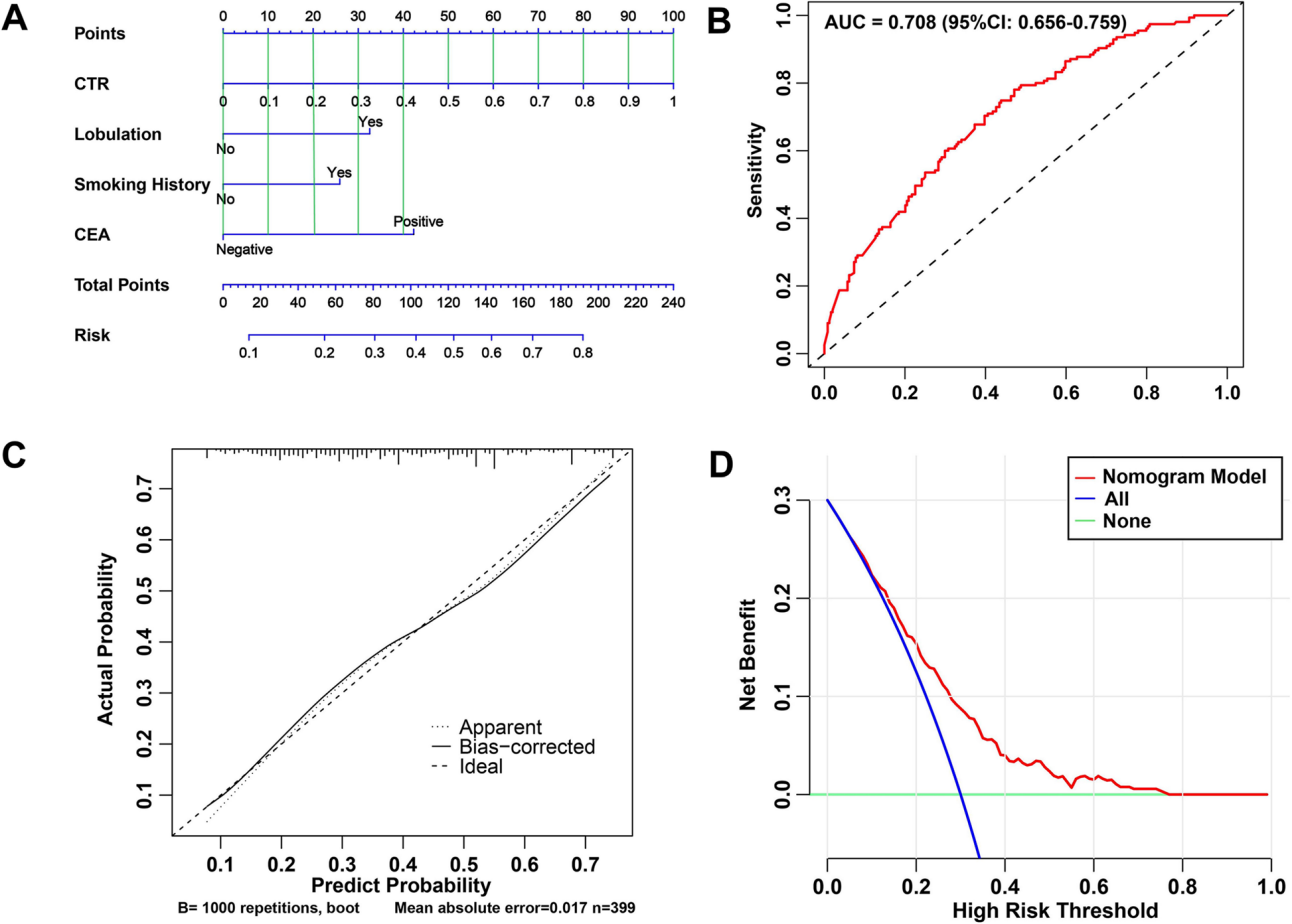
Development and internal validation of prediction model using training cohort. **a** The nomogram prediction model for grade 3 tumors in clinical stage I LUAD. **b** ROC curve for the nomogram (AUC = 0.708). **c** The calibration curve of the nomogram. **d** The DCA curve of the nomogram. AUC: area under the receiver operating characteristic curve; CTR: consolidation-to-tumor ratio; Tdmax: maximum tumor diameter; Positive CEA levels represent greater than 4.7 ng/ml and negative levels represent less than 4.7 ng/ml

**Fig. 4:**
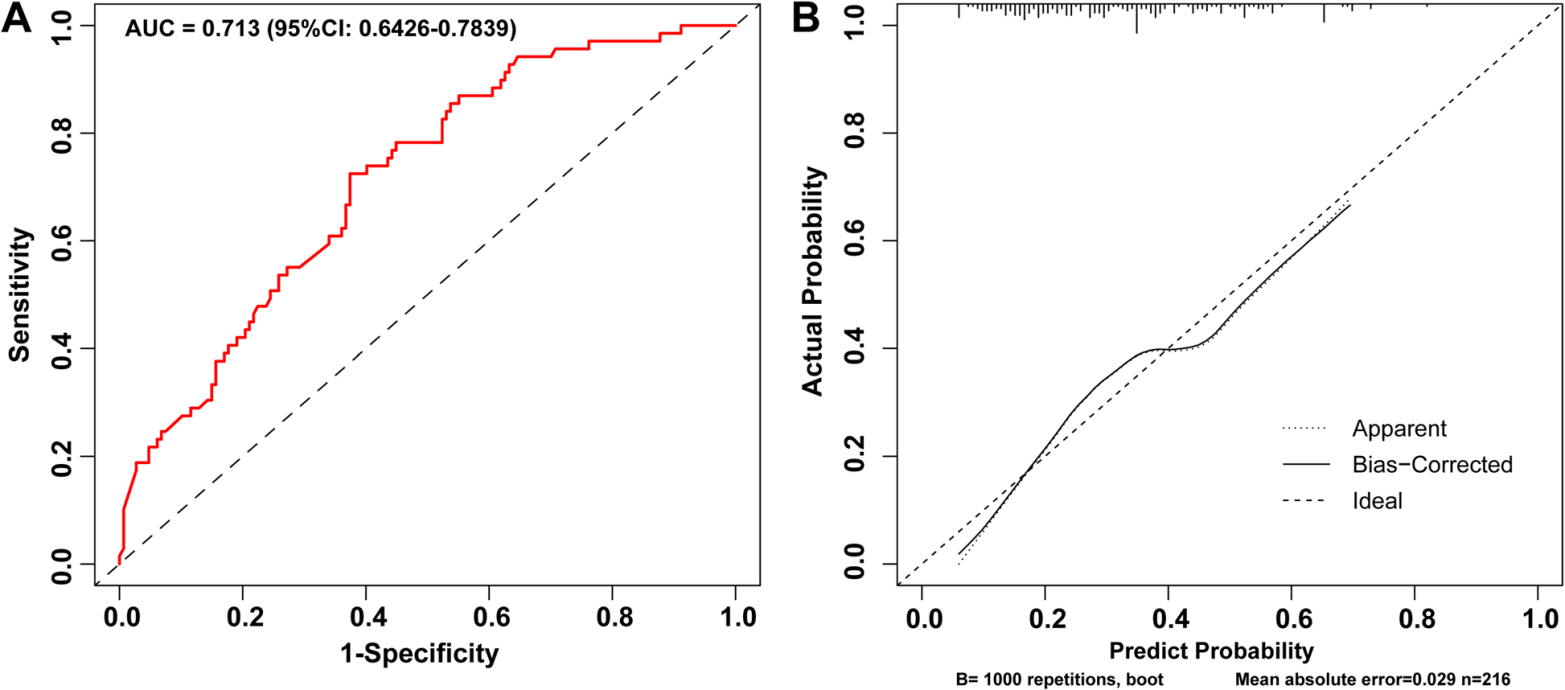
External validation of prediction model using validation cohort. **a** ROC curve of the nomogram (AUC = 0,713). **b** The calibration curve of the nomogram. AUC: area under the receiver operating characteristic curve

A predictive model was constructed based on the four independent risk factors and visualized with a nomogram (Fig. 3a). Internal validation was performed by bootstrapping, and external validation was completed in an independent patient cohort (Validation set mentioned in the method section) to verify the results. The model had good discrimination (AUC = 0.708, 95% CI: 0.6563–0.7586, Fig. 3b), and calibration curves showed that the predicted probability of the model was very close to the actual probability (Fig. 3c). The Hosmer–Lemeshow test result was χ^2^ = 7.052 (*P* = 0.531), indicating that the model underwent a proper calibration. DCA showed that the nomogram prediction model obtained a better net clinical benefit from the intervention decision than the baseline model when the risk was 0.1–0.78 (Fig. 3d). The model discrimination was good in the external validation cohort, showing proper predictive ability (AUC = 0.713, 95% CI: 0.6426–0.7839, Fig. 4a). However, the calibration curve by the bootstrap method showed moderate consistency (Fig. 4b).

## Discussion

Recently, the IASLC Pathology Committee integrated high-grade subtypes and predominant pathological subtypes to develop a three-tiered grading system for invasive nonmucinous LUAD [1]. The widely used grading systems in the past were based on major subtypes, including the Architectural grading system and Sica’s grading system, but the disadvantage of these systems was that the poor prognostic patterns, especially micropapillary patterns, that only contained a small portion of the "nonpredominant" amounts of the tumor were also associated with adverse outcomes [4, 18, 19]. Therefore, since the proposal of the new IASLC grading system, many researchers have conducted validation studies, all of which have shown excellent ability in differentiating prognosis [9, 10, 20, 21]. Kagimoto et al. first validated the IASLC grading system and found that the system had a good stratification effect on RFS in patients with pathological stage 0 or I LUAD [20]. Meanwhile, Rokutan-Kurata et al. revealed the utility of the new grading system in determining the prognosis of patients with invasive adenocarcinoma in a large cohort in Japan and found excellent interobserver agreement (k = 0.94) [9]. Subsequently, Hou et al. revealed the prognostic significance of the IASLC grading system in a multicenter Chinese cohort and provided clinical value to guide treatment decisions regarding adjuvant chemotherapy [21]. Ultimately, the 2021 WHO Classification of Thoracic Tumors continued to recommend recording the percentage of histological patterns of invasive nonmucinous LUAD and applying a formal grading system utilizing these features [2].

Previous studies have demonstrated that the presence of high-grade pathological components leads to poor prognosis; grade 3 LUAD has the highest content of high-grade patterns, and its prognosis is significantly worse than that of non-grade 3 LUAD [1, 5, 19, 22]. Watanabe et al. found that the presence of micropapillary increased the risk of early recurrence, and the micropapillary components were associated with poor prognosis even after complete resection in patients with stage I LUAD [22]. Solid and papillary patterns in stage IB LUAD were associated, if not predominant linked, with poor prognosis, according to Zhao et al. [23]. In addition, complex glandular patterns and cribriform patterns, as new high-grade pathological components, had once been included in the high-grade acinar subtype, but many studies have confirmed that they were more aggressive than acinar structures and were associated with poor prognosis [5–8]. Kadota et al. demonstrated that the presence of cribriform patterns (> 10%) was an independent predictor of recurrence and later found that cribriform patterns were an independent factor of poor prognosis and should be distinguished from acinar subtypes in patients with resected LUAD [6, 7]. Moreira et al. found that the DFS of tumors mainly composed of complex glandular patterns was similar to that of high-grade tumors (*P* = 0.932) but significantly worse than that of low-grade and intermediate-grade tumors (*P* = 0.0025), suggesting that complex glandular patterns had important prognostic value [5]. Furthermore, Yoshida et al. indicated that IASLC grade 3 tumors were an independent factor for poor prognosis in patients with LUAD [11]. Thus, grade 3 adenocarcinomas based on the IASLC grading system should be primarily identified and treated with greater caution.

**Fig. 5:**
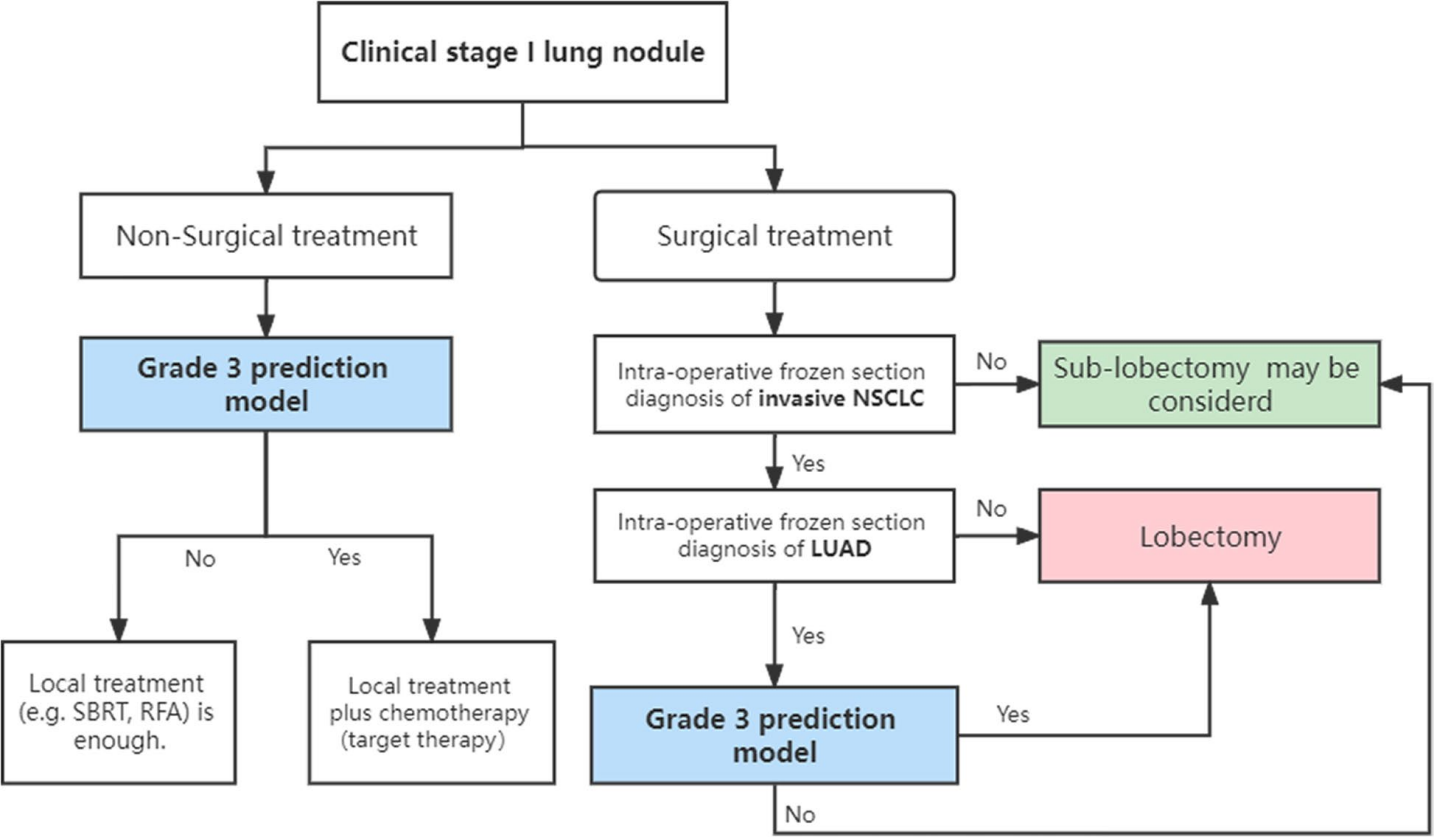
A pre-treatment decision diagram based on grade 3 prediction model

Pathological grading is often used as a reference for adjuvant treatment after surgery, but its application in surgical and nonsurgical treatment decisions has not been developed [2, 24, 25]. Previous studies have shown that lobectomy is safer for LUAD with high-grade pathological components [14, 15, 26]. Nitadori et al. showed that LUAD patients with greater than or equal to 5% of the micropapillary patterns treated by sublobectomy had a higher risk of recurrence than similar patients treated by lobectomy, suggesting that sublobectomy may not be appropriate for LUAD patients containing any micropapillary components [15]. Song et al. pointed out that lobectomy was recommended for invasive LUAD with pN0 and ≤ 1 cm, and wedge resection can obtain similar oncological effects only for lepidic or acinar predominant tumors [26]. In addition, the survival analysis results in this study suggested that the RFS of the lobectomy group was better than that of the sublobectomy group in grade 3 adenocarcinomas. Therefore, lobectomy is recommended for grade 3 LUAD based on the above results. However, surgical decision-making relies on adequate evaluation of preoperative biopsy specimens or intraoperative frozen sections, whereas treatment options for nonsurgical patients also need to be supported by biopsy pathology results, but these measures have limited ability to evaluate pathological grades [13, 16, 17]. Based on the poor prognosis of grade 3 LUAD, evaluating the pathological grades before treatment, especially grade 3 tumors, can benefit the survival of relevant patients. In this study, the pretreatment prediction model of grade 3 tumors was established and visualized by a nomogram, which can intuitively reflect the probability of grade 3. We proposed a treatment decision diagram based on the grade 3 prediction model, which can provide individual assessment of grade 3 possibilities for each LUAD patient before treatment, thereby optimizing treatment decisions (Fig. 5).

The predictors in the model included CTR, lobulation, smoking history, and CEA level, and similar results have been shown in previous studies [27–30]. Chen et al. defined CTR > 0.5 as radiological invasiveness and used it to construct a radiomics model for predicting micropapillary and solid components, with the sensitivity and specificity reaching 90.00% and 45.21%, respectively [27]. According to the study of Hu et al., there are significant differences in CT morphology between different pathological subtypes, except the acinar and papillary subtypes [28]. Moreover, Yi et al. found that smoking was closely related to micropapillary and solid tumors [29]. The CEA level was proved to be highest in solid-predominant adenocarcinoma and to be independently associated with the presence of solid and micropapillary components, as reported by Li et al. [30]. In addition, the indicators of the nomogram prediction model were readily available in clinical practice, which is suitable for routine application in clinical work.

## Limitation

There were some shortcomings in this study, including the following: (i) This study was a single-center retrospective study with a relatively small sample size, and a further prospective study with a large sample size is required in the future; (ii) Temporal validation was selected for external validation in this study, which indicated that multicenter external validation is required in the future to further improve transportability and generalization; and (iii) The clinical and imaging variables included in this study were limited, and in the future, they need to be supplemented and expanded by deep learning, radiomics and other models.

## Conclusion

Through univariate and multivariate analyses, we found that CTR, lobulation, smoking history and CEA level were independent risk factors for grade 3 tumors and established a pretreatment prediction model for grade 3 tumors. According to the results of survival analysis, the RFS of patients undergoing lobectomy in the grade 3 group was superior to that of patients undergoing sublobectomy. Therefore, in line with previous studies, we suggest that high-risk patients with grade 3 disease screened out by the model should undergo lobectomy, and we have developed a treatment decision diagram that provides convenient conditions for clinical application.

### Supplementary Information

**Additional file 1: Supplementary Table 1.** Baseline characteristics of training and validation set.

## Data Availability

Due to the privacy of patients participating in the study, the datasets generated or analyzed during the current study are not publicly available, but available from the corresponding author upon reasonable request.
