# Supplementary material for: A pretreatment prediction model of grade 3 tumors classed by the IASLC grading system in lung adenocarcinoma
Source: BMC Pulm Med. 2023 Oct 7;23:377. doi: 10.1186/s12890-023-02690-3 (PMC10559613; doi:10.1186/s12890-023-02690-3)
Supplement: Supplementary file 1 — Additional file 1: Supplementary Table 1. Baseline characteristics of training and validation set. [file 12890_2023_2690_MOESM1_ESM.docx]

**Supplementary Table 1 Baseline characteristics of training and validation set**

| Characteristics | Training set  (n = 399) | Validation set  (n = 216) | *P* Value |
| --- | --- | --- | --- |
| Grading, n (%) |  |  |  |
| Grade 3 | 155(38.8) | 69(31.9) | 0.089 |
| non-Grade 3 | 244(61.2) | 147(68.1) |  |
| Age (years), median (P25, P75) | 63(58,67) | 61(54,66) | 0.018 |
| Gender, n (%) |  |  |  |
| Male | 183(45.9) | 85(39.4) | 0.12 |
| Female | 216(54.1) | 131(60.6) |  |
| Preoperative symptoms, n (%) |  |  |  |
| Yes | 86(21.6) | 38(17.6) | 0.242 |
| No | 313(78.4) | 178(82.4) |  |
| Smoking history, n (%) |  |  |  |
| Yes | 123(30.8) | 54(25.0) | 0.128 |
| No | 276(69.2) | 162(75.0) |  |
| CEA (ng/ml), n (%) |  |  |  |
| Positive (＞4.7) | 84(21.1) | 35(16.2) | 0.146 |
| Negative (0-4.7) | 315(78.9) | 181(83.8) |  |
| Tumor location, n (%) |  |  |  |
| Central | 11(2.8) | 4(1.9) | 0.592 |
| Peripheral | 388(97.2) | 212(98.1) |  |
| Lobular location, n (%) |  |  |  |
| Middle/Lower lobe | 165(41.4) | 82(38.0) | 0.413 |
| Upper Lobe | 234(58.6) | 134(62.0) |  |
| Tdmax (cm), median (P25, P75) | 2.132(1.598,2.638) | 1.99(1.455,2.368) | <0.001 |
| CTR, median (P25, P75) | 0.635(0.415,0.857) | 0.619(0.405,0.808) | 0.159 |
| Spiculation, n (%) |  |  |  |
| Yes | 236(59.1) | 100(46.3) | 0.002 |
| No | 163(40.9) | 116(53.7) |  |
| Pleural retraction, n (%) |  |  |  |
| Yes | 241(60.4) | 123(56.9) | 0.405 |
| No | 158(39.6) | 93(43.1) |  |
| Vacuole, n (%) |  |  |  |
| Yes | 115(28.8) | 65(30.1) | 0.741 |
| No | 284(71.2) | 151(69.9) |  |
| Lobulation, n (%) |  |  |  |
| Yes | 207(51.9) | 102(47.2) | 0.27 |
| No | 192(48.1) | 114(52.8) |  |
| Air bronchogram, n (%) |  |  |  |
| Yes | 106(26.6) | 55(25.5) | 0.766 |
| No | 293(73.4) | 161(74.5) |  |
